# A Critical Introduction to Sex and Nature in the Anthropocene

**DOI:** 10.1215/22011919-9962915

**Published:** 2022-11-01

**Authors:** Sarah Bezan, Ina Linge

**Affiliations:** Department of English and Related Literature, Leverhulme Centre for Anthropocene Biodiversity, University of York, UK; Department of Languages, Cultures and Visual Studies, University of Exeter, UK

## Neanderthal Sex in Space

It’s been over a year, but we are still choking on the fumes of billionaire Jeff Bezos’s dick-rocket voyage. On Twitter and on late night television programs, Bezos’s self-indulgent joyride in a phallic spacecraft was easy comedic fodder. “Space bout to get fucked,” one Twitter user quipped, while Jon Stewart and other comedians compared Bezos’s suborbital flight to the shuttle used by supervillain Dr. Evil in the film *Austin Powers: The Spy Who Shagged Me.*^2^ Still others commented on the relatively short duration of the space flight itself (ten minutes and ten seconds), likening it to premature ejaculation.^3^ The entire venture was over before most of the public had any idea that it had occurred, yet much of the sentiment about it was entirely predictable. For many, the enterprise was not an act of inspiration but a gratuitous show of American exceptionalism in which the expanse of space was rendered *extraterrestrial nullius:* the neocolonial frontier. Much like the 1969 landing of the first man on the moon, which Jack Halberstam describes as significant because it designated “the end of something, perhaps the end of man, the end of white men in particular, the end of the human,”^4^ Bezos’s flight through space prompts considerations of a future earth that is imagined to be rewilded and returned to its prehuman state, while an advantaged offshoot of humanity grows anew elsewhere.

If it appears as if the future is in forward motion as much as it is in retrograde, it is because visions of the unfolding Anthropocene epoch are plagued by these kinds of atavistic mythologies. In an era of unprecedented ecological crises and accelerating losses in species biodiversity, the future often looks backward. Archaeologist Rebecca Wragg Sykes’s recent best-selling book, *Kindred: Neanderthal Life, Love, Death, and Art*, for instance, outlines how the twenty-first century “love affair” with the extinct Neanderthal is a story that “extends into our far future.”^5^ As an object lesson in survival beyond extinction, the Neanderthal has ostensibly come back into vogue. Indeed, high-profile Harvard geneticist George Church once mused dreamily about putting Neanderthal clones into outer space. “Neanderthals might think differently than we do,” Church told a German press in 2013. He continued: “We know that they had a larger cranial size. They could even be more intelligent than us. When the time comes to deal with an epidemic or getting off the planet or whatever, it’s conceivable that their way of thinking could be beneficial.”^6^ To achieve this feat of technoscientific resurrection, Church suggested that a reverse-engineered Neanderthal clone would need to be implanted into “the uterus of an extraordinarily adventurous female.”^7^ This uterus would in effect become a time machine, with “DNA itself [acting as] a kind of spaceship.”^8^ While this inverse exercise in genomic time traveling has not yet been seriously attempted, the image of a gestating Neanderthal clone that carries extinct prehumanity into a posthuman future remains a potent, but undoubtedly chauvinistic and reprocentric, fantasy.

Added to this fantasy are the racial politics implied due to the place that the Neanderthal occupies within Western imaginaries. In *Superior: The Return of Race Science*, science journalist Angela Saini writes that “kinship has been established between Europeans and Neanderthals” as a result of recent archaeological findings and genomic sequencing that link Neanderthal DNA with living humans.^9^ Given the potential to problematically blur the boundaries between the sociopolitical dimensions of race and the cultural-natural ontologies of species, this warped idea of kinship suggests that Neanderthals are no longer perceived to be “evolutionary dead-ends” as they were in the nineteenth and twentieth centuries.^10^ Instead, they are now—at least in Church’s technoscientific vision—looked upon as a back-up hominid species in possession of attributes that supposedly align with Western values: intelligence, compassion, innovation. To this end, the Neanderthal clone could be regarded as an ideal safeguard of European civilization, preserving the white, straight, and colonial ideals that are thought to be under the threat of obsolescence.

Church’s fantasy of reanimated Neanderthals puttering around in outer space invites us to consider the ways in which sex and nature remain contested terms. While plotting out the discrete origin point and future trajectory of the human is a spurious endeavor at best, we have much to learn from retracing and interrogating the intellectual histories of sex and nature. We argue, in fact, that emerging research in Anthropocene studies needs more rigorous historicization if it is to confront regressive thinking about sex and nature and to instead offer a broader array of possibilities for inclusive kinship and more-than-human relations. This special issue aims to intervene in precisely this way: by uniting historians of sexuality with scholars in the environmental humanities, the contributors to this issue step on and off a revolving time machine, pinpointing particular instances across the nineteenth, twentieth, and twenty-first centuries that illuminate meaningful shifts in our understanding of sex, gender, and the environment, which can in turn be brought to bear upon new ecological realities and sociopolitical conditions.

## Historicizing Sex, Gender, and the Environment in the Anthropocene

The work of this special issue begins by drawing together scholars of gender and sexuality studies with scholars in the field of environmental humanities, whose work intersects but has yet to fully integrate mutually beneficial practices and approaches. On the one hand, we consider how concepts of sex, gender, and sexuality variously shape the ways in which individuals, groups, or cultures understand, relate to, or are excluded from nature and the environment both historically and in the present moment. On the other, we examine how environmental histories are conditioned by, but also unravel, stultifying and exclusionary categorizations of sex and nature that have been met with resistance at various moments in time. This dual approach is important, given that, as Juno Salazar Parreñas and Nicole Seymour point out in their afterword to this issue, the meaning of *ecology*—a term coined by German zoologist Ernst Haeckel in 1866—is a system of thought that is in flux across the nineteenth, twentieth, and twenty-first centuries, and is often put to use in ways that negate queer modes of being and relating. In the wake of studies of the Anthropocene (a concept that is itself subject to interrogation), the field of queer ecologies plays a vital role in tracing the antecedent threads of sex and nature. Doing so presents us with an opportunity to understand how changing environmental conditions might renew or resist the historical contingencies that gave rise to these meanings.

As other queer ecologists and ecofeminists have already shown, there has been a bracketing out of gender and sexuality in environmental humanities scholarship since the term environmental humanities emerged with the inauguration of *Environmental Humanities* in 2012. This is despite the fact that, as Jennifer Mae Hamilton and Astrida Neimanis argue, feminist ideas and concepts were foundational to the emergence of the field of environmental humanities.^11^ Nonetheless, some theoretical approaches to the environmental humanities highlight the important contribution of the humanities in analyzing different forms of meaning-making as both cognitive and normative, and how such meaning is projected onto the natural environment.^12^ Yet a number of these approaches fail to account for the fact that one of the major lenses through which cultures and individuals produce meaning is through gender and sexuality. This is particularly true for Anglophone environmental history, which, as environmental historians Morgan and Cook suggest, is marred by the lack of a sustained research focus on women, gender, and sexuality.^13^

Scholars in queer ecology have tackled this lack of interdisciplinary exchange between queer theory and the history of sexuality as well as environmental history and environmental humanities. In their field-defining work *Queer Ecologies: Sex, Nature, Politics, Desire*, Catriona Sandilands and Bruce Erickson consider the “junctures at which lgbtq (lesbian/gay/bisexual/transgender/queer) and environmental politics (both defined broadly) intersect.”^14^ In developing what they call queer ecology, they aim “to probe the intersections of sex and nature with an eye to developing a sexual politics that more clearly includes considerations of the natural world and its biosocial constitution, and an environmental politics that demonstrates an understanding of the ways in which sexual relations organize and influence both the material world of nature and our perceptions, experiences, and constitutions of that world.”^15^ Sex and nature are not only mutually entangled, as outlined by Erickson and Sandilands, but also structurally similar. As Timothy Morton argues in his guest column for *PMLA* on queer ecology, “ideologies of Nature are founded on inside-outside structures that resemble the boundaries heterosexism polices.”^16^ The decade since the publication of *Queer Ecologies* has seen an ever-burgeoning field of intersectional ecological scholarship by queer, feminist, anti-colonial, and BIPOC scholars who, building on these structural analyses of sex and nature laid out by Erickson and Sandilands, are “branching out to redefine understandings of the ‘natural’ technosciences in a time of extinction and multiscalar change.”^17^ These include, but are certainly not limited to, critical analyses of Blackness, racialization, and animalization in the works of Zakiyyah Iman Jackson, Alexander G. Weheliye, Chelsea Mikael Frazier, and Bénédicte Boisseron, along with scholarship on the relationship between ecologies and race/ethnicity by Neel Ahuja, Jasbir K. Puar, Mel Y. Chen, and Claire Jean Kim.^18^ Indigenous perspectives on more-than-human relations, animality, and settler colonialism by Billy-Ray Belcourt (Driftpile Cree Nation) and Kim TallBear (Sisseton Wahpeton Oyate) have likewise outlined more inclusive and expansive categories of relationality, sexuality, and kinship structures.^19^ Our contributors are working alongside this assembly of scholars to reflect on the persistence of white, cis-normative, reprocentric, and heterosexual framings of Anthropocene pasts and futures. In doing so, they ask: if sex, gender, and sexuality and their politics and histories can shape our understanding of the environment or how women, BIPOC, and LGBTQ+ people can be variously excluded from these categories, what place do we imagine for sex, gender, and sexuality in the Anthropocene?

In addition to queer ecologies, new material feminist approaches—from Alaimo and Hekman’s *Material Feminisms* to Karen Barad’s agential realism^20^ —have modeled ways of rethinking the spatial, temporal, and corporeal boundaries of sex and nature. In *Anthropocene Feminism*, contemporary feminist critics reflect on the place of feminist and queer thought in the Anthropocene age. In the introduction, Richard Grusin proposes a question that outlines the contribution of feminism to Anthropocene thought: “Insofar as early feminism begins with a critique of nature, a critique of the idea that gender differences were biological, that gender was natural, how does feminism address the definition of the human as a geological force, the embrace of the naturalness of ‘man’?”^21^ This historicization of feminist thought could be thought differently, considering that women’s rights activists, including sexologists, in the early twentieth century recognized the power of grounding gender and sexuality in nature.^22^ As Grusin points out, nature has always been a focal point for feminist thought, and Anthropocene feminism emphasizes how queer and feminist theories can offer an alternative to “the too often unquestioned masculinist and technonormative approach to the Anthropocene taken by technoscientists, artists, humanists, or social scientists.”^23^

Building on the work of groundbreaking volumes like *Queer Ecologies* and *Anthropocene Feminism*, one of the provocations we offer in this special issue is that the scale of the Anthropocene obscures and essentializes the detailed, entangled, and complex histories that give meaning to the human, the nonhuman, and the environment. As we have seen from Bezos’s dick-rocket voyage and Church’s technoscientific fantasy of Neanderthal clones, fears and anxieties about the consequences of the Anthropocene often trigger debates about the future of all human and nonhuman life on the planet. However, the massive scale of the Anthropocene obscures the fact that, as outlined by geographer Kathryn Yusoff in *A Billion Black Anthropocenes or None*, the *anthropos* (or humanity) of the Anthropocene is not a homogeneous or unified whole.^24^

Yusoff outlines a number of potential origin points for the Anthropocene that have been proposed by scholars across the natural and social sciences, as well as within the humanities: from 13,800 years ago to 1610 (the time of the Columbian exchange), 1800 (the Industrial Revolution, marked by the invention of the steam engine), or the 1950s (which mark the Great Acceleration and use of nuclear isotopes). However, selecting a single origin point for the Anthropocene epoch has, as Liana Chua and Heather Fair argue in *The Cambridge Encyclopedia of Anthropology*, “political and socio-economic repercussions.”^25^ They write that “depending on the starting date that is chosen, particular processes will come to be held responsible for our current planetary predicament. This will suggest certain avenues for action, and foreclose others.”^26^ Yusoff responds to some of the political and socioeconomic dimensions of Anthropocene origin stories. By focusing on the racial politics and colonial legacies of the Anthropocene, Yusoff argues that the process of deciding upon an origin point for this epoch is a matter of power relations and agencies. Yusoff’s treatise aims to challenge the idea of Man as an organizing principle for the Anthropocene by identifying a “need to desediment the social life of geology.”^27^ As with the issues inherent to collapsing the boundaries between biologically differentiated hominid species and socially constructed racial categories, this kind of desedimentary methodology recognizes not merely an origin point but the continued presence of racial and colonial legacies that constitute the Anthropocene as an epoch-in-the-making.^28^ More importantly, how we imagine the Anthropocene and the place of human and nonhumans in a sustainable future therefore depends on the racialized, colonial, gendered, and variously constructed ways in which the human, the nonhuman, and the environment were upheld in the past.

## The Queer Child and the Birth Pangs of Anthropocene Futures

Jeremy Davies’s *The Birth of the Anthropocene* describes the “present environmental crisis” as evidence of “the birth pangs of a new epoch.”^29^ Outlining the extent to which the rigidly masculinist logics of Anthropocene discourse are wrapped up in the trope of a ravaged Mother Earth, these figurations of historical and environmental change in the Anthropocene suggest that its progenies are synonymous with its futures. Indeed, out of this maternal trope comes, tentatively and precariously, the figure of the Child. The imperative to protect the Child, however, often reveals the extent to which our hopes for and projections of the future are steeped in racial, colonial, and sexist narratives. As Nicole Seymour points out in her analysis of an environmental advocacy ad depicting a white male child in a suburban environment that has been threatened by mercury poisoning, environmental hopes “frame environmental degradation as a threat not just to a particular child, or even many children, but to a particular way of life … link[ing] sentimental heterosexism and environmentalism.”^30^ As scholars of color and Indigenous scholars have argued, Black and Indigenous people and especially those living in the Global South have long been living with the consequences of climate change in the Anthropocene.^31^ Furthermore, queer and trans people, especially queer and trans people of color, often experience heightened suffering as a result of natural disasters resulting from climate change. In the UK, almost a quarter of homeless youth are LGBTQ+ and are therefore particularly vulnerable to weather events caused by climate change.^32^ LGBTQ+ people often face discrimination when accessing support, for example in shelters, and experience difficulties when trying to cross borders to escape conflict and disaster. As a response to this, the grassroots collective Wretched of the Earth challenged environmental organizations like Extinction Rebellion to think critically about class, racialization, and capitalism in tandem with sexuality and gender, highlighting the need to both queer and decolonize climate activism.^33^

Seymour rightly points out that fully understanding the implication of environmental visions of the future and the ideologies underlying these visions requires more than a twopronged analysis via queer theory and environmental humanities. Instead, we are in need of a broader and more diverse set of queer-ecological methods and approaches to sex and nature in the Anthropocene. Drawing on queer theory to further develop a queer ecological methodology, Seymour notes the tensions inherent in environmental humanities’ focus on the future and the rejection of future-oriented thinking in some queer theoretical approaches. In *No Future*, queer theorist Lee Edelman advocates for a rejection of “reproductive futurism,” organized around the Child as a product and proponent of heteronormativity seeking the survival of the social order and communal relations. As with Church’s gestating Neanderthal clone, the Child here becomes the “emblem of futurity’s unquestioned value.”^34^ Edelman, however, stridently opposes this valued reproductive futurism with the “project of a queer oppositionality,” which rejects the very logic of oppositionality and, in doing so, rejects “every substantialization of identity” as well as “history as a linear narrative.”^35^

Edelman’s rejection of futurity has been questioned by queer of color critique, notably José Esteban Muñoz’s work, which highlights that Edelman’s critique only works when thinking exclusively about the white child. Muñoz cautions that “it is important not to hand over futurity to normative white reproductive futurity.”^36^ Furthermore, scholars of queer ecology have argued that the precarious future is no longer a fiction—nor a technoscientific fantasy—but a certainty.^37^ Sarah Ensor criticizes that in both “the futurism of the traditional environmentalist and the annihilating stance of the antisocial queer theorist … the present and the future become mutually delimiting realms.”^38^ In this special issue we argue that we cannot turn toward a future of Anthropocene scale without understanding the complex set of terms and categories invoked variously to define nature, environment, sexuality, and gender in at least the recent past. Whatever the future holds, it is rooted in the past and present moment.

The theoretical implications of queer negativity can be important for environmental humanities in two ways: First, a rejection of futurity, or any viable political future, can help to unsettle the inevitable narrative that ties environmental thinking and climate change to a predicted universal future and instead return to the inequalities of the here and now. Second, we are invited to dwell on the complexities and confusions of identities. This appreciation of the slipperiness of categories that are always formed in oppositional ways allows us to understand the centrality of socially constructed terms and categories in projections of the future. “Queer” is thought in opposition to the figure of the Child as “universalized subject.”^39^ The urgency with which the Anthropocene and the detrimental consequences of climate change are perceived risks relying on such universalizing figures. Queer theory, in contrast, offers a consideration of irreparable divisions and of negative affects such as loss, loneliness, violence, or disagreement. This allows us to begin to account for the “stubborn particularity” of queerness that resists generalization in the context of the massive scale of the Anthropocene.^40^

We propose that this “stubborn particularity” of queerness and the stubborn particularity with which concepts of sex, gender, and sexuality circulate, shape, and are variously embedded in and resistant to ideologies offer a fruitful intervention into the reductive scale of the Anthropocene. In particular, in this special issue we are interested in the ways in which sex—as a short form encompassing gender, sexuality, and queerness—and nature—or the nonhuman and the environment—are entangled and mutually constitutive in stubborn particularity. We ask, how do intersections of sex and nature produce, block, or queer knowledge production? To imagine a sustainable future and sustainable relationship between humans and their natural environment in the Anthropocene, too little time is spent on reflecting both the complexity of the human as well as the historically contingent understanding of nature and its entanglements with other concepts of the human. Changing concepts of the natural were key to historical sexual knowledge production during sexual modernity, but scholars of the history of sexuality often treat nature as a “conceptual whipping boy,” dismissing it as rigid, normalizing, and an antithesis to the cultural realm of gender and sexuality.^41^ Here, the conceptual and historical links between sex and nature are forcibly broken, and nature is defined in oppositional terms to the realm of culture. This obscures the fact that nature is conceptually slippery and contrary.

Together, our contributors demonstrate that today’s environmental conditions are historically shaped by ideas and practices of sexual nature but also present sites of theorization that reform our understanding of nineteenth, twentieth, and twenty-first century sex, gender, and sexualities. These articles in turn advance critical frameworks for thinking beyond sex and subjectivity (i.e., the environmental expressions of sex), the mediation of human and nonhuman erotics (such as that of human bodies and bodies of water), reprocentrism (evolutionary understandings of reproductive sex, futurity, and “fitness”), and queer and ecofeminist paradigms that challenge the naturalization of sexual politics (alt-right, neo-fascist, dystopian). In this special issue, then, these complex, contradictory, and nuanced understandings of the relationship between sex and nature show that just as the *anthropos* is neither unified nor monolithic, neither can we assume that there is an all-inclusive understanding of nature or the environment that is equally or universally accommodating.

Our articles in this special issue are necessarily selective in their choice of case studies and historical foci, which has the effect of countering the essentialized or abstract narratives that are characteristic of single-origin Anthropocene thinking. There is good reason why several articles in this collection turn toward the second half of the nineteenth century and first decades of the twentieth century and in particular bring the cultural, political, and onto-epistemological legacies of whiteness into focalized critique. During this period in white Western history, sex, gender, and sexuality were brought under a conceptual framework of sexual subjectivity. Sexuality in this context was understood as arising variously from nature or nurture and as situated both in body and mind.^42^ Medico-scientific discourses were increasingly interested in investigating the causes and symptoms of gender and sexual diversity, culminating in the study of sexual sciences and adjacent fields such as psychoanalysis, endocrinology, and genetics. These broadly scientific approaches were linked to social movements concerned for the health of the individual and the health of a nation, including social hygiene movements and eugenics. What was considered healthy and normal was highly ideological, as sex during this period of sexual modernity was also reframed as almost entirely for white women and men. At the same time, scholars in the history of sexuality and in the history of sexology more specifically have shown how sexual scientists variously drew on concepts of nature and the natural to either condone or legitimize sexual diversity.^43^ We do not suggest that the intersection between sex and nature originates in the late nineteenth and early twentieth century, nor do we argue that a critique of whiteness alone can fully outline the painful legacies that surround the normativization of sex and nature as categories. Likewise, we recognize the limits of this special issue’s focus on primarily white texts and cultural media, which despite its self-reflexive modality and critique of whiteness, risks perpetuating the knowledge frameworks it seeks to cross-examine (see the section titled “Are Queer Ecologies White?” in this issue’s afterword). As we carefully approach these historical archives and cultural representations of whiteness, the articles in this special issue show that during this period concepts of gender, sexuality, race, nature, and the environment emerged in ways that are still dominant and relevant today, and which necessitate continued, and urgent, critique.

The articles in this issue show that recognizing retrograde histories as well as resistances to such histories gives us a foundation on which we can build more critically engaged futures. By thinking through critical debates about sex and nature, our return to the history and development of these terms seeks to add new vocabularies and dialogues for responding to radical shifts in environmental conditions. As work continues across the fields of history of sexuality and the environmental humanities, we urge the continued growth of practices, aesthetics, methodologies, and conceptual histories of sex and nature that unravel the development of these entangled categories across both Western and non-Western knowledge frameworks. The articles in this special issue work to guide us toward new methods and practices that might reinvestigate these imperfect histories. Looking ahead, we hope that the articles collected here can offer an impetus to imagine alternative kinship structures for the future, renewed ways of relating, and a path toward queer and nonnormative ways of being in the Anthropocene.

SARAH BEZAN is postdoctoral research associate in perceptions of biodiversity change at the University of York, UK. Her research examines the entangled cultural and ecological dimensions of species loss and revival.

INA LINGE is lecturer in German in the Department of Languages, Cultures and Visual Studies at the University of Exeter, UK. Her research in queer German studies, environmental humanities, and medical humanities investigates early twentieth-century sexual knowledge production as a collaborative endeavor between the arts and medical and natural sciences.

## Footnotes

2 Amber Ruffin (@ambermruffin), “Space bout to get fucked,” Twitter, July 20, 2021, 2:24 p.m., https://twitter.com/ambermruffin/status/1417475558871547911.

3 Nubian American Foundation and Alliances N.A.F.A. (@TGIFrani), “Aussie hosts can’t keep straight face over Jeff Bezos’ #penisrocket,” June 12, 2021, 8:41 p.m., https://twitter.com/TGIFrani/status/1403799658208456707.

4 Halberstam, “In/Human-Out/Human,” 239.

5 Sykes, *Kindred*, 14−15.

6 *Der Spiegel International*, “Interview.”

7 *Der Spiegel International*, “Interview.”

8 *Der Spiegel International*, “Interview.”

9 Saini, *Superior*, 36.

10 Saini, *Superior*, 36.

11 Hamilton and Neimanis, “Composting Feminisms.”

12 Castree, “Environmental Humanities.”

13 Morgan and Cook, “Gender and Environment.”

14 Mortimer-Sandilands and Erickson, “Genealogy,” 2.

15 Mortimer-Sandilands and Erickson, “Genealogy,” 5.

16 Morton, “Guest Column,” 274.

17 Vaughn, Guarasci, and Moore, “Intersectional Ecologies,” 17.6. See also Seymour, “Black Lives,” in which Seymour outlines how work in queer ecology needs to center BIPOC and emerging scholars.

18 Jackson, *Becoming Human;* Weheliye, *Habeas Viscus;* Frazier, “Black Feminist Ecological Thought”; Boisseron, *Afro-Dog;* Ahuja, *Planetary Specters;* Puar, *Terrorist Assemblages;* Chen, *Animacies*; Kim, *Dangerous Crossings*.

19 Belcourt, “Animal Bodies.” On critical polyamory, see Kim TallBear, *Unsettle* (blog), https://kimtallbear.substack.com/.

20 Alaimo and Hekman, *Material Feminisms;* Barad, “Posthumanist Performativity.”

21 Grusin, “Introduction,” ix.

22 Leng, *Sexual Politics*.

23 Grusin, “Introduction,” x.

24 Yusoff, *Billion Black Anthropocenes*.

25 Chua and Fair, “Anthropocene,” 3.

26 Chua and Fair, “Anthropocene,” 3.

27 Yusoff, *Billion Black Anthropocenes*, 54, 58−59.

28 Yusoff, *Billion Black Anthropocenes*, 62.

29 Davies, *Birth of the Anthropocene*, 2.

30 Seymour, *Strange Natures*, vii.

31 Shiva, *Soil, Not Oil*, 34.

32 Bhandal and Horwood, *LGBTQ+ Youth Homelessness Report*.

33 Wretched of the Earth, “Open Letter.”

34 Edelman, *No Future*, 4.

35 Edelman, *No Future*, 4.

36 Muñoz, “Cruising the Toilet,” 365.

37 Chaudhuri, “Sun’ll be Hotter Tomorrow”; Ensor, “Spinster Ecology,” 409. Cited in Evans, “Fantastic Futures?”

38 Ensor, “Spinster Ecology,” 428.

39 Edelman, *No Future*, 11.

40 Edelman, *No Future*, 6.

41 Seymour, *Strange Natures*, 4.

42 Sutton, *Sex between Body and Mind*, 2.

43 LaFleur, *Natural History;* Milam, *Looking for a Few Good Males;* Cryle and Downing, “Natural and the Normal”; Brooks, “Darwin’s Closet.”

## References

[R1] Ahuja Neel (2021). Planetary Specters: Race, Migration, and Climate Change in the Twenty-First Century.

[R2] Alaimo Stacy, Hekman Susan (2008). Material Feminisms.

[R3] Barad Karen (2003). Posthumanist Performativity: Toward an Understanding of How Matter Comes to Matter. Signs.

[R4] Belcourt Billy-Ray (2015). Animal Bodies, Colonial Subjects: (Re)Locating Animality in Decolonial Thought. Societies.

[R5] Bhandal Jo, Horwood Matt (2021). The LGBTQ+ Youth Homelessness Report.

[R6] Boisseron Bénédicte (2018). Afro-Dog: Blackness and the Animal Question.

[R7] Brooks Ross (1871). Darwin’s Closet: The Queer Sides of the Descent of Man. Zoological Journal of the Linnean Society.

[R8] Castree Noel, Richardson Douglas, Castree Noel, Michael F, Goodchild Audrey Kobayashi, Liu Weidong, Marston Richard A (2021). The International Encyclopedia of Geography.

[R9] Chaudhuri Una (2014). Sun’ll be Hotter Tomorrow: Growing Up with Climate Chaos. Resilience.

[R10] Chen Mel Y (2012). Animacies: Biopolitics, Racial Mattering, and Queer Affect.

[R11] Chua Liana, Fair Hannah (2019). The Cambridge Encyclopaedia of Anthropology.

[R12] Cryle Peter, Downing Lisa (2010). The Natural and the Normal in the History of Sexuality Special issue. Psychology and Sexuality.

[R13] Davies Jeremy (2016). The Birth of the Anthropocene.

[R14] Der Spiegel International (2013). Interview with George Church: Can Neanderthals Be Brought Back from the Dead?.

[R15] Edelman Lee (2004). No Future: Queer Theory and the Death Drive.

[R16] Ensor Sarah (2012). Spinster Ecology: Rachel Carson, Sarah Orne Jewett, and Nonreproductive Futurity. American Literature.

[R17] Evans Rebecca (2017). Fantastic Futures? Cli-fi, Climate Justice, and Queer Futurity. Resilience.

[R18] Frazier helsea Mikael, Atmos (2020). Black Feminist Ecological Thought: A Manifesto.

[R19] Grusin Richard, Grusin Richard (2017). Anthropocene Feminism.

[R20] Halberstam Jack (2015). In/Human-Out/Human. GLQ.

[R21] Hamilton Jennifer Mae, Neimanis Astrida (2018). Composting Feminisms and Environmental Humanities. Environmental Humanities.

[R22] Jackson Zakiyyah Iman (2020). Becoming Human: Matter and Meaning in an Antiblack World.

[R23] Kim Claire Jean (2015). Dangerous Crossings: Race, Species, and Nature in a Multicultural Age.

[R24] LaFleur Greta (2018). The Natural History of Sexuality in Early America.

[R25] Leng Kirsten (2018). Sexual Politics and Feminist Science.

[R26] Milam Erika Lorraine (2010). Looking for a Few Good Males: Female Choice in Evolutionary Biology.

[R27] Morgan Ruth A, Cook Margaret (2021). Gender and Environment Special issue. International Review of Environmental History.

[R28] Mortimer-Sandilands Catriona, Erickson Bruce, Mortimer-Sandilands Catriona, Erickson Bruce (2010). Queer Ecologies: Sex, Nature, Politics, Desire.

[R29] Morton Timothy (2010). Guest Column: Queer Ecology. PMLA.

[R30] Muñoz José Esteban (2007). Cruising the Toilet: LeRoi Jones/Amiri Baraka, Radical Black Traditions, and Queer Futurity. GLQ.

[R31] Puar Jasbir K (2007). Terrorist Assemblages: Homonationalism in Queer Times.

[R32] Saini Angela (2020). Superior: The Return of Race Science.

[R33] Shiva Vandana (2016). Soil, Not Oil: Climate Change, Peak Oil, and Food Insecurity.

[R34] Seymour Nicole (2013). Strange Natures: Futurity, Empathy, and the Queer Ecological Imagination.

[R35] Seymour Nicole, DeWitt Jessica (2020). Succession: Queering the Environment.

[R36] Sutton Katie (2019). Sex between Body and Mind: Psychoanalysis and Sexology in the German-Speaking World, 1890s-1930s.

[R37] Sykes Rebecca Wragg (2020). Kindred: Neanderthal Life, Love, Death, and Art.

[R38] Vaughn Sarah E, Guarasci Bridget, Moore Amelia (2021). Intersectional Ecologies:Reimagining Anthropology and Environment. Annual Review of Anthropology.

[R39] Weheliye Alexander G (2014). Habeas Viscus: Racializing Assemblages, Biopolitics, and Black Feminist Theories of the Human.

[R40] Wretched of the Earth (2019). An Open Letter to Extinction Rebellion. Red Pepper.

[R41] Yusoff Kathryn (2018). A Billion Black Anthropocenes or None.

